# ERD: a fast and reliable tool for RNA design including constraints

**DOI:** 10.1186/s12859-014-0444-5

**Published:** 2015-01-28

**Authors:** Ali Esmaili-Taheri, Mohammad Ganjtabesh

**Affiliations:** 1 Department of Computer Science, School of Mathematics, Statistics, and Computer Science, College of Science, University of Tehran, Tehran, Iran; 2 Laboratoire d’Informatique (LIX), Ecole Polytechnique, Palaiseau CEDEX, 91128 France

**Keywords:** RNA Structure, RNA design, Energy constraint

## Abstract

**Background:**

The function of an RNA in cellular processes is directly related to its structure. The free energy of RNA structure in another important key to its function as only some structures with a specific level of free energy can take part in cellular reactions. Therefore, to perform a specific function, a particular RNA structure with specific level of free energy is required. For a given RNA structure, the goal of the RNA design problem is to design an RNA sequence that folds into the given structure. To mimic the biological features of RNA sequences and structures, some sequence and energy constraints should be considered in designing RNA. Although the level of free energy is important, it is not considered in the available approaches for RNA design problem.

**Results:**

In this paper, we present a new version of our evolutionary algorithm for RNA design problem, entitled ERD, and extend it to handle some sequence and energy constraints. In the sequence constraints, one can restrict sequence positions to a fixed nucleotide or to a subset of nucleotides. As for the energy constraint, one can specify an interval for the free energy ranges of the designed sequences. We compare our algorithm with INFO-RNA, MODENA, NUPACK, and RNAiFold approaches for some artificial and natural RNA secondary structures and constraints.

**Conclusions:**

The results indicate that our algorithm outperforms the other mentioned approaches in terms of accuracy, speedup, divergency, nucleotides distribution, and similarity to the natural RNA sequences. Particularly, the designed RNA sequences in our method are much more reliable and similar to the natural counterparts. The generated sequences are more diverse and they have closer nucleotides distribution to the natural one. The ERD tool and web server are freely available at http://mostafa.ut.ac.ir/corna/erd-cons/.

## Background

Ribonucleic acids play fundamental roles in cellular processes and their functions are directly related to their structures. The function of an RNA is highly dependent on its three-dimensional conformation which is referred to as RNA tertiary structure. Since the prediction or experimental determination of tertiary structure is very difficult, so many works focus on the problems associated with the RNA secondary structure.

An important problem in the RNA research area is the RNA inverse folding, in which, the secondary structure of an RNA is given and the goal is to find a proper sequence that folds into the given structure. The RNA inverse folding problem can be used to design non-coding RNAs, which are involved in gene regulation, chromosome replication and RNA modification [1,2]. The designed sequences are also applicable to the construction of ribozymes and riboswitches, which may be used as drugs and therapeutic agents in research [3], or for building self-assembling structures from small RNA molecules in nano-biotechnology [4]. In the RNA inverse folding problem, there is an exponential number of sequences to be considered as candidates for the solution [5-7]. It is also suggested that the RNA inverse folding problem may be NP-Hard, i.e., the time required to find an exact global solution grows exponentially [8]. Therefore, the heuristic search methods are widely used to address this problem [3,4,9-14].

RNAinverse, available as a part of the Vienna RNA package, is an original approach to solve this problem [12]. The second algorithm, called RNA-SSD which is developed by Andronescu et al. (2004), tries to minimize the structural distance via recursive stochastic local search [10]. Busch and Backofen (2006) proposed another algorithm based on dynamic programming and local search, called INFO-RNA [3]. This algorithm consists of two steps. In the first step, it generates an initial sequence using dynamic programming. In the second step, it uses a stochastic local search method to improve the quality of the initial sequence. Genetic algorithm is also used to solve the RNA inverse folding problem, both for RNA secondary structure [15,16] and pseudoknotted structures [16,17]. In [18], a dynamic programming approach (NUPACK) is employed for designing the RNA sequence that is intended to adopt a target secondary structure at equilibrium. They formulated the sequence design problem as an optimization problem with the goal of reducing the ensemble defect.

A Constraint Programming (CP) approach, entitled RNAiFold, is presented to solve the RNA inverse folding problem. This approach allows a wide range of design constraints to be specified [7]. It also introduces a Large Neighborhood Search (LNS) approach which allows larger instances at the cost of losing completeness, while retaining the advantages of meeting design constraints (motif, GC-content, etc.). IncaRNAtion [19] implements a novel algorithm based on weighted sampling techniques [20] that enables user to control explicitly the GC-content of the solution. This functionality is useful because wild-type sequences within living organisms often present medium or low GC-content, presumably to offer better transcription rates and/or structural plasticity. RNAdesign is another tool for designing RNA sequences that fold into multiple target structures [21]. It uses the graph coloring techniques and heuristic local optimization algorithm to find sequences whose energy landscapes are dominated by the prescribed conformations.

In this paper, we extend our original Evolutionary RNA Design (ERD) algorithm [5] to address its previous limitations and to offer new functionalities. First, we consider the RNA inverse folding problem satisfying some sequence constraints. These constraints can restrict certain positions to a fixed nucleotide or to a fixed subset of nucleotides. Next, a new functionality for bounding the free energy of generated sequences over the given structure to a specified interval is presented. This energy constraint is essential, since only some structures with a specific level of energy can take part in certain biological reactions. The new extended ERD tool can also be used to design the RNA elements that include conserved nucleotides, which are essential for binding proteins.

## Methods

In this section, we briefly introduce our evolutionary algorithm for designing an RNA sequence that folds into a given target structure [5]. Any RNA secondary structure can be uniquely decomposed into its structural components (stems, hairpin loops, internal loops, bulge loops, multi-loops, and external loop), each having a different length. By employing the natural RNA sequences, we first construct the pools of RNA sub-sequences corresponding to different components with different lengths. Using these pools, we then construct an initial RNA sequence which is compatible with the given target structure. After that, the target structure is hierarchically decomposed into smaller sub-structures. This decomposition is performed in positions where the multi-loops occur. Finally, we use an evolutionary algorithm to improve the quality of the sub-sequences corresponding to the decomposed sub-structures. The ERD tool has been implemented in *C* programming language to be consistent with the Vienna RNA package and to benefit from the faster execution of the compiled code in this language. The details of the above-mentioned steps are presented in the following subsections.

### Pools reconstruction

In order to design RNA sequences similar to the natural ones, we use an existing database of natural RNA sequences (namely, STRAND [22]) to construct the pools of RNA sub-sequences. To do this, for each sequence in this database, the *fold* method of the Vienna RNA package is executed to obtain its secondary structure. Then, this structure is decomposed into its structural components and the sub-sequences of the same type and length are gathered into the same pool.

### Construct the initial sequence

To be consistent with the other parts of our algorithm, specially when we improve the quality of the sub-sequences corresponding to the components, we assign a compatible RNA sequence to the given target structure. To do this, the target structure is decomposed into its structural components and a sub-sequence is randomly picked from the corresponding pool based on the type and length of each component. These sub-sequences are then assembled to produce a compatible sequence for the target structure. It should be mentioned that this initial sequence is not guaranteed to be folded into the target structure and therefore it should be considered for further improvements. In addition, the initial sequence should satisfies the sequence and free energy constraints, if they are specified. Therefore, if the initial sequence violates some constraints, another initial sequence is constructed. This step is repeated up to 1000 times.

In addition to the sequence constraints, the minimum and maximum energy ranges can be specified for the generated sequences over the given structure. The default maximum energy range is 0 and default minimum energy range is −*∞*. After generating the initial sequence, its thermodynamic free energy over the target structure is evaluated by employing the *e* *n* *e* *r* *g* *y*_*o* *f*_*s* *t* *r* *u* *c* *t* *u* *r* *e* method (available as a part of the Vienna RNA Package). If this free energy does not belong to the specified energy ranges, another initial sequence is generated to satisfy the energy constraint. This step is repeated up to 1000 times.

### Hierarchical structure decomposition

Since any folding algorithm requires at least *O*(*n* ^3^) operations, improving the whole initial sequence will increase the overall running time of any heuristic algorithm. On the other hand, decomposing the target structure into its structural components produces many small components and increases the number of iterations, and consequently increases the running time. In order to speed up the running time of our algorithm, we employ an intermediate decomposition scheme to decompose the target structure into its sub-structures. This decomposition is done in positions where the multi-loops occur in the given target structure. Let the given structure consist of *k* multiloops *M*_1_,*M*_2_,…,*M*_*k*_. For each *p* (1≤*p*≤*k*), let *M*_*p*_ contain *q* closing base pairs, say ${{i^{1}_{p}}}.{{j^{1}_{p}}},\; {{i^{2}_{p}}}.{{j^{2}_{p}}},\; \cdots,\;{{i^{q}_{p}}}.{{j^{q}_{p}}}$. We define an order over the closing bases as follows:

$${i^{a}_{p}}.{j^{a}_{p}} < {i^{b}_{p}}.{j^{b}_{p}} \Longleftrightarrow {i^{a}_{p}} < {i^{b}_{p}} $$

**Figure 1:**
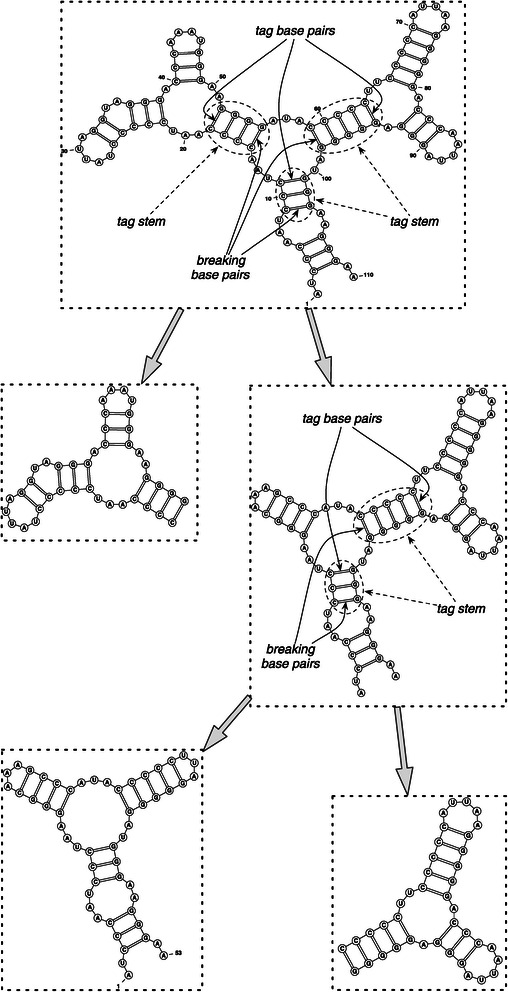
**Hierarchical decomposition [** 5 **].** The hierarchical decomposition of target structure into its sub-structures.

Based on this ordering, the minimum closing base pair in each multi-loop is called *tag base pair* and the stem containing a tag base pair is called *tag stem*. Now, the minimum base pair (in tag stem with respect to the previously defined order) is marked as a *breaking base pair*. The tag base pairs, as well as the breaking base pairs are shown in Figure 1, which is drawn by VARNA [23]. If several breaking base pairs are available, the one whose resulting sub-structures have almost equal lengths is chosen. The process of decomposing the given structure is performed recursively to yield the hierarchy over the sub-structures. The initial compatible sequence is also decomposed exactly in the same positions with respect to the corresponding sub-structure (See [5] for details).

### Evolutionary algorithm

After constructing the pools of RNA sub-sequences as well as the initial sequence, and decomposing the given structure into sub-structures, we employ an evolutionary algorithm to improve the quality of the initial RNA sequence. The first step in our evolutionary algorithm is the construction of the initial population. To do this, we use the *fold* method (available as a part of the Vienna RNA Package) over the initial sequence as input to determine its Minimum Free Energy (MFE) secondary structure. The predicted structure may differ from the target structure in some positions. Then we choose the components containing these positions of differences and for each of them we replace its corresponding sub-sequence with another sub-sequence from the appropriate pool, regarding its type and length. These new sequences are considered as the initial population. To evaluate the quality of the sequences in the resultant population, several steps are taken in our algorithm. These sequences are first evaluated by employing the *energy_of_structure* method (available as a part of the Vienna RNA Package) to determine their thermodynamic free energy over the target structure. Then they are sorted increasingly according to their energy values. Among them, the three best sequences (with lowest energies) are selected for further evaluations. The structures of these three sequences are determined by employing the *fold* method. Next, the Hamming distance between the target structure and the three selected structures, as well as the best structure found, are calculated. Again, the best three of them are chosen as a basis for generating the next population. The best one is also stored as the best solution till now. It should be noted that, in our evolutionary algorithm, we do not have crossover operation and the mutation operates in component level (not in nucleotide level). The termination condition in our algorithm (without constraints) is either finding a solution with the Hamming distance equal to zero or continuing the above processes for at most 250 iterations (in this case, the final best solution is reported).

In an extension to [5], the ERD tool can handle a set of user-defined constraints on the generated sequences. These constraints have to be satisfied during the execution of the algorithm. The evolutionary step in the ERD is the most appropriate place to consider the constraints, i.e the sub-sequences in the current population are modified in such a way that satisfy the given constraints.

In addition to the sequence constraints, the minimum and maximum energy ranges can be specified for the generated sequences over the given structure. If an interval is specified for the energy value, instead of choosing the most stable sequence of the current population as the best one, a sequence that has the least energy difference with the center of the specified energy interval is selected as the best one. In this case, the ERD terminates when either the number of iterations reaches its maximum or a sequence is found where its structure has zero Hamming distance to the target structure, it satisfies the sequence constraints, and its energy value over the target structure belongs to the specified interval. Therefore, the ERD tool returns the best-found RNA sequence satisfying both the sequence and energy constraints.

## Results and discussion

As shown in [5], ERD approach (without constraints) proceeds better and more rapidly than the other existing approaches, specially for longer structures. It has been examined over the natural and artificial RNA structures and the results indicate that ERD has higher success count as well as lower computational time. It also produces RNA sequences with wider energy ranges, i.e. the generated sequences are distributed more diverse in the solution space. The average energy values of the generated sequences by ERD over the target structures are closer to those of natural sequences, compared with the other approaches. This helps us to select an RNA sequence whose minimum free energy is closer to the natural counterparts. The Boltzmann probabilities of the designed sequences are also closer to those of natural sequences. The distribution of nucleotides and base pairs is also analyzed for the generated sequences and the distribution of the ERD generated sequences is much closer to the natural distribution of nucleotides and base pairs, compared with the other approaches.

### Dataset for benchmark constraints

In order to test the accuracy and reliability of our algorithm with constraints, we employ two different datasets. The first one is the dataset which is used by RNA-SSD [10] (dataset *A*). This dataset contains 8 natural structures of length between 65 and 583 nucleotides. The biological description of these structures are presented in Table 1. The second dataset is chosen from [24] which contains 12 structures of length 178 and 176 nucleotides (dataset *B*), consisting of artificial miRNA structures that are published in [25] (see Table 2).

**Table 1:** **Biological description of the sequences and structures in dataset** ***A***

**Index**	**Description**	**Length (nt)**
A1	Minimal catalytic domains of the hairpin	65
	ribozyme satelite RNA of the Tobacco ringspot	
A2	U3 snoRNA 5’ domain from Chlamydomonas	79
	reinhardtii, in vivo probing	
A3	H.marismortui 5S rRNA	122
A4	VS Ribozyme from Neurospora mitochondria	166
A5	XS1 Ribozyme, Bacillus subtilis P RNA based	314
	ribozyme	
A6	Homo Sapiens RiboNuclease P RNA	340
A7	S20 mRNA from E. coli	372
A8	Group II intron ribozyme D135 from Saccharomyces	583
	cerevisiae mitochondria

**Table 2:** **Biological description of the sequences and structures in dataset** ***B***

**Index**	**Description**	**Length (nt)**
B1	pre-amiR-lfy-1	178
B2	pre-amiR-lfy-2	178
B3	pre-amiR-white-1	178
B4	pre-amiR-white-2	178
B5	pre-amiR-ft-1	178
B6	pre-amiR-ft-2	178
B7	pre-amiR-trichome	178
B8	pre-amiR-mads-1	178
B9	pre-amiR-mads-2	178
B10	pre-amiR-yabby-1	178
B11	pre-amiR-yabby-2	178
B12	pre-miRNA	176

Since no sequence constraints are available for the structures in dataset *A*, three different sets of constraints are randomly generated for this dataset. In the first set of constraints, only 10*%* of positions in each structure are randomly selected and a random nucleotide (with uniform distribution) is assigned to each selected position and considered as sequence constraints (dataset *A*−*C*10). In the second and third sets of constraints, respectively 20*%* and 30*%* of positions are randomly selected, fixed, and considered as constraints (datasets *A*−*C*20 and *A*−*C*30, respectively). For the structures in dataset *B*, their corresponding sequence constraints are available, in which the natural RNA sequences of the structures are employed to fix some positions. The third dataset (dataset *C*) contains 408 sequences of length between 36 and 1509 nucleotides that are selected from *R* *f* *a* *m* 11. To this end, the first sequence in each block of 940 sequences in *R* *f* *a* *m* 11 is selected.

The results of our algorithm over the above mentioned datasets are compared with the results of four other approaches, namely INFO-RNA, MODENA, NUPACK, and RNAiFold. Different measures are employed in our comparisons to determine the accuracy and reliability of the competitor approaches. All the results (except for RNAiFold) are obtained by a computer with Intel Core2Duo (2.26 *G* *H* *z*) CPU, having 2*G* *B* of memory, and running Linux Ubuntu (11.04) as operating system. The Vienna RNA Package (version 1.8.5) along with the Turner free energy parameters [26] are employed in all approaches. NUPACK is the only approach that uses the ensemble defect as a fitness function, where the threshold value is considered 0.01. As for MODENA, the default parameters (50 for both population size and iterations) are utilized. The results of RNAiFold are either obtained from its web server (for datasets *A* and *B*) or generated by a powerful cluster (for dataset *C*). Since the hardware specification to run the RNAiFold is different, the time is not provided for this method in our comparisons.

For each structure and its related constraint sequence in datasets *A* and *B*, all the mentioned approaches are executed 50 times, where the time limit of 1800 seconds is considered for each execution. The success count (*SC*) indicates how often each approach successfully designs an RNA sequence (among 50 executions) for each given structure and constraint. The expected time (*E*_*T*_) indicates how much time is required for successfully designing an RNA sequence for each structure and it is calculated as follows:

(1) $$ {E_{T}} = \frac{{Total\;Execution\;Time}}{{SC}}.  $$

All the mentioned approaches are executed one time for the structures and their corresponding sequences in dataset *C*. The time limit of 3600 seconds are considered for each execution. Here, the *SC* indicates how many sequences are successfully designed for 408 structures in this dataset. The expected summation of energy distance (*E*_*ED*_) indicates how much energy value of successfully designed sequences of any approach is different from the energy value of natural sequence and it is calculated as follows:

(2) $$ {E_{ED}} = \frac{{\sum \mid E_{sd}-E_{n}\mid}}{{SC}},   $$

where *E*_*sd*_ indicates the energy value of successfully designed sequence and *E*_*n*_ indicates the energy value of it corresponding natural sequence.

The accuracy and speed comparisons of our algorithm with respect to the other approaches are presented in Tables 3, 4 and 5, respectively for the constructed datasets of random constraints *A*−*C*10, *A*−*C*20, and *A*−*C*30. The *∞* sign in these tables indicates that the corresponding approach could not design an appropriate sequence for the given structure and constraint in the period of the execution time limit. The best results are also indicated in bold face in these tables. Since MODENA returns all correctly generated sequences as results (among 50 sequences in its final population), the *E*_*T*_ of MODENA is calculated by considering one execution time as its total execution time, in order to be fair in our comparison. As it is mentioned in Tables 3, 4 and 5, our algorithm performs much better than MODENA in all cases, and it is better than INFO-RNA and RNAiFold in most of the cases. Compared with NUPACK, our algorithm is superior specially in terms of computational time.

**Table 3:** **Results for dataset** ***A-C*** **10**

	**INFO-RNA**		**MODENA**		**NUPACK**		**RNAiFold**	**ERD**	
**Index**	***SC***	***E***_***T***_	***SC***	***E***_***T***_	***SC***	***E***_***T***_	***SC***	***SC***	***E***_***T***_
A1	38	**0.15**	47	33.09	50	12.56	50	50	0.17
A2	50	**0.03**	39	35.85	50	4.38	50	50	0.08
A3	32	1.08	40	49.05	46	76.88	50	**50**	**0.82**
A4	50	**0.33**	16	114.78	50	172.61	50	50	0.93
A5	4	1558.55	42	283.96	35	1740.54	50	**50**	**172.74**
A6	4	2612.28	14	287.76	20	3704.05	0	**50**	**34.30**
A7	0	*∞*	22	335.26	0	*∞*	50	**50**	**175.35**
A8	19	**536.49**	0	*∞*	0	*∞*	0	**25**	1097.67

The success count (*SC*) and expected time (*E*_*T*_) comparison between the existing approaches for dataset *A-C*10, including 10% of sequence constraints. The ***∞*** represents no result and the bold faces indicate the best results.

**Table 4:** **Results for dataset** ***A-C*** **20**

	**INFO-RNA**		**MODENA**		**NUPACK**		**RNAiFold**	**ERD**	
**Index**	***SC***	***E***_***T***_	***SC***	***E***_***T***_	***SC***	***E***_***T***_	***SC***	***SC***	***E***_***T***_
A1	32	0.23	30	32.06	50	9.24	50	50	**0.11**
A2	50	**0.03**	45	37.57	50	5.80	50	50	0.06
A3	47	**0.20**	45	61.52	45	28.97	50	**50**	0.77
A4	0	*∞*	0	*∞*	0	*∞*	0	0	*∞*
A5	2	4130.43	38	305.61	5	17698.03	0	**40**	**154.68**
A6	12	592.57	39	309.57	50	836.15	50	50	**13.06**
A7	0	*∞*	0	*∞*	0	*∞*	0	0	*∞*
A8	0	*∞*	0	*∞*	**45**	**1133.91**	0	0	*∞*

The success count (*SC*) and expected time (*E*_*T*_) comparison between the existing approaches for dataset *AC*20, including 20% of sequence constraints. The ***∞*** represents no result and the bold faces indicate the best results.

**Table 5:** **Results for dataset** ***A-C30***

	**INFO-RNA**		**MODENA**		**NUPACK**		**RNAiFold**	**ERD**	
**Index**	***SC***	***E***_***T***_	***SC***	***E***_***T***_	***SC***	***E***_***T***_	***SC***	***SC***	***E***_***T***_
A1	42	**0.10**	40	31.65	50	8.80	50	50	0.12
A2	50	**0.04**	41	37.52	50	3.35	50	50	0.05
A3	20	**3.97**	7	60.67	34	164.47	0	**41**	4.74
A4	0	*∞*	0	*∞*	**50**	**479.95**	0	0	*∞*
A5	0	*∞*	0	*∞*	0	*∞*	0	**5**	**2472.85**
A6	0	*∞*	0	*∞*	0	*∞*	0	0	*∞*
A7	0	*∞*	0	*∞*	0	*∞*	0	0	*∞*
A8	32	**52.13**	43	1124.62	25	1059.20	33	**50**	124.56

The success count (*SC*) and expected time (*E*_*T*_) comparison between the existing approaches for dataset *A-C*30, including 30% of sequence constraints. The ***∞*** represents no result and the bold faces indicate the best results.

The same comparisons between the existing approaches for dataset *B* are presented in Table 6. The ERD-EC column in this table relates to the results of ERD when the energy constraint is also specified. The energy interval for the generated sequences for dataset *B* is considered as the natural energy value ±10. As it is understood, the computational time is increased when the energy interval is specified. Again, the superiority of our approach is concluded form this table. Table 7 indicates that the average energy values of the generated sequences by ERD over the given structures are closer to the natural energies, compared with the other approaches. Also, when the energy constraint is applied, more reliable sequences are generated by ERD. This helps us to select an RNA sequence whose secondary structure has free energy value closer to that of natural counterparts. Comparing with the other methods, INFO-RNA is superior in generating sequences that have lower minimum free energy structure.

**Table 6:** **Results for dataset** ***B***

	**INFO-RNA**		**MODENA**		**NUPACK**		**RNAiFold**	**ERD**		**ERD-EC**	
**Index**	***SC***	***E***_***T***_	***SC***	***E***_***T***_	***SC***	***E***_***T***_	***SC***	***SC***	***E***_***T***_	***SC***	***E***_***T***_
B1	29	7.16	46	131.05	50	22.28	0	50	**2.42**	50	74.20
B2	26	12.01	40	143.55	50	24.29	0	50	**2.24**	50	53.52
B3	25	10.50	44	139.07	50	10.58	50	50	**2.73**	50	48.05
B4	27	12.59	36	171.93	50	23.63	0	50	**2.45**	50	77.71
B5	25	12.10	48	169.66	50	11.79	50	50	**3.13**	50	67.24
B6	22	14.42	40	185.99	50	11.87	0	50	**2.87**	50	77.20
B7	44	**2.21**	37	173.37	50	24.58	0	50	2.38	50	57.86
B8	26	12.68	45	168.02	50	15.23	50	50	**2.48**	50	55.80
B9	31	11.27	41	173.59	50	11.38	50	50	**2.54**	50	85.09
B10	27	9.62	43	171.63	50	13.50	50	50	**2.44**	50	71.05
B11	44	**2.82**	40	167.91	50	11.75	0	50	3.13	50	61.63
B12	27	14.53	47	175.10	50	28.69	0	50	**2.53**	50	70.12

The success count (*SC*) and expected time (*E*_*T*_) comparison between the existing approaches for dataset *B*, including natural sequence constraints. The best results are indicated in bold face.

**Table 7:** **The average free energy values of sequences generated for dataset** ***B***

**Index**	**Natural**	**INFO-RNA**	**MODENA**	**NUPACK**	**RNAiFold**	**ERD**	**ERD-EC**
B1	-72.69	-152.16	-117.89	-112.41	−	-105.87	**-87.34**
B2	-72.69	-151.71	-120.34	-115.04	−	-106.49	**-84.85**
B3	-75.19	-155.22	-115.10	-109.54	-108.30	-108.36	**-87.45**
B4	-69.29	-149.49	-120.21	-113.08	−	-103.32	**-84.39**
B5	-75.19	-154.02	-105.83	-111.39	-104.20	-109.97	**-89.81**
B6	-71.49	-152.47	-115.60	-107.05	−	-107.63	**-88.61**
B7	-75.49	-156.88	-115.35	-118.80	−	-112.18	**-91.39**
B8	-69.69	-152.14	-113.89	-110.18	-99.61	-104.70	**-81.71**
B9	-72.19	-149.95	-116.41	-111.26	-111.40	-106.91	**-93.78**
B10	-73.49	-155.33	-123.21	-111.02	-107.40	-110.94	**-88.88**
B11	-76.79	-156.54	-123.35	-114.59	−	-114.78	**-89.56**
B12	-74.49	-154.24	-113.75	-115.51	−	-110.36	**-92.38**

The average free energy (*A*_*E*_) values of sequences generated by different approaches and the corresponding natural energy values for dataset *B*. ERD-EC is related to the ERD when the energy constraint is specified. The closest energy values to the natural ones are indicated in bold face.

Two important questions are, 1) Which approach can generate divers sequences for the given target structure? and 2) Which approach can generate sequences similar to natural one? To answer these questions, the similarity between the generated sequences as well as the similarity of them to the corresponding natural sequence must be calculated. To do this, the *needle* software from the *EMBOSS* is employed [27] to calculate the similarities. EMBOSS (the European Molecular Biology Open Software Suite) is a free open source software analysis package specially developed for the needs of the molecular biologist. The software automatically copes with data in a variety of formats and even allows transparent retrieval of sequence data from the web. Also, as extensive libraries are provided with the package, it is an appropriate platform to allow other scientists to develop and release software. *needle* uses the Needleman-Wunsch alignment algorithm to find the optimum alignment (including gaps) of two sequences along their entire length [28,29]. The algorithm uses a dynamic programming method to ensure the alignment is optimum, by exploring all possible alignments and choosing the best one. A scoring matrix is provided for every possible residue or nucleotide match. For each approach, the expected similarity between the generated sequences for different dataset are presented in Table 8. Since the success counts are almost different for all approach, the expected similarity for the generated sequences by each approach (*E*_*SA*_) is calculated as follows:

**Table 8:** **The expected similarity of the generated sequences**

**Dataset**	**INFO-RNA**	**MODENA**	**NUPACK**	**RNAiFold**	**ERD**
*A*−*C*10	74.97	75.09	47.91	98.01	**45.22**
*A*−*C*20	72.76	76.93	51.01	97.40	**50.44**
*A*−*C*30	80.20	78.30	60.15	97.37	**53.50**
*B*	80.55	70.69	**48.96**	97.12	53.57

The expected similarity (*E*_*SA*_) between the generated sequences by each approach on different datasets. The lower similarities are indicated in bold face.

(3) $$ {E_{SA}} = \frac{1}{{|D|}}\sum\limits_{I \in D} {\frac{{\sum\limits_{i = 1}^{SC(I)} {\sum\limits_{j = i + 1}^{SC(I)} {similarity({S_{i}},{S_{j}}})} }}{{\frac{{SC(I)(SC(I) - 1)}}{2}}}},  $$

where *D* is the employed dataset, *I* indicates a structure of *D*, *S* *C*(*I*) represents the success count for the structure *I*, *S*_*i*_ (*S*_*j*_) indicates the *i*th (*j*th) successfully designed sequence for the structure *I*, and *s* *i* *m* *i* *l* *a* *r* *i* *t* *y*(*S*_*i*_,*S*_*j*_) calculates the similarity between *S*_*i*_ and *S*_*j*_. As it is understood from Table 8, the generated sequences by ERD are less similar (more divers) comparing with the other competitors.

Also, the expected similarity of the generated sequences by each approach to the corresponding natural sequence (*E*_*SN*_) is calculated as follows:

(4) $$ E_{SN}=\frac{\sum\limits_{i=1}^{SC}similarity(S_{A_{i}},S_{N_{i}})}{SC},  $$

where $S_{A_{i}}$ is the *i*th successfully designed sequence for each approach and $S_{N_{i}}$ is the corresponding natural sequence in dataset C. The calculated values of *E*_*SN*_ for each approach are shown in Table 9. As it is obvious, ERD is superior in time, accuracy, and similarity to the natural sequences comparing with the other approach. The ERD-EC in this table is related to the results of ERD when the energy constraint (±10*%* of the natural energy value) is specified. The expected consequences of applying the energy constraint are: 1) increasing the expected time, 2) decreasing the success count, 3) generating sequences that have lower expected energy distance, and 4) generating sequences that are more similar to the natural counterpart.

**Table 9:** **Results for dataset C**

**Approach**	***E***_***T***_	***SC***	***E***_***ED***_	***E***_***SN***_
INFO-RNA	43.79	32	54.86	28.69
MODENA	165.86	395	27.05	35.73
NUPACK	483.41	336	20.21	38.91
RNAifold	1888.32	272	29.09	31.12
ERD	**6.11**	**401**	13.22	39.44
ERD-EC	217.99	386	**7.94**	**39.88**

The comparison of expected time (*E*_*T*_), success count (*SC*), expected energy distance (*E*_*ED*_), and expected similarity to the natural sequences (*E*_*SN*_) between the existing approaches for dataset C. ERD-EC is related to the ERD when the energy constraint is specified. The *SC* indicates how many sequences are successfully designed. The best results are indicated in bold face.

The final test we have done over the generated sequences is the distribution of nucleotides. We would like to see which method produce RNA sequences with distribution closer to the natural distribution of each nucleotide. To do this, the natural distribution of each nucleotide appeared in different structural components is calculated. Then the generated sequences of each method are analyzed and the distribution of each nucleotide in different structural components are calculated and presented in Table 10. As it is shown in this table, the distribution of nucleotides for the sequences generated by ERD is much closer to the natural distribution of nucleotides with respect to the other approaches. For example, all mentioned methods (except ERD and NUPACK) employ almost CG base pairs in generating sub-sequences corresponding to the stems. Here, ERD regards the natural distribution of nucleotides comparing with NUPACK. Also, MODENA and RNAiFold employ almost the nucleotides A for generating the sub-sequences corresponding to the loops, whereas ERD, INFO-RNA, and NUPACK employ the other nucleotides as well.

**Table 10:** **The nucleotides distribution in the generated sequences for all datasets**

	**Paired**				**Unpaired**					**Total**			
	**AU**	**GC**	**GU**		**A**	**C**	**G**	**U**		**A**	**C**	**G**	**U**
Natural	0.39	0.49	0.12		0.38	0.19	0.17	0.26		0.27	0.22	0.25	0.26
ERD	**0.31**	**0.62**	**0.07**		0.41	**0.19**	**0.17**	0.23		**0.26**	**0.26**	**0.27**	**0.21**
INFO-RNA	0.05	0.90	0.05		**0.37**	0.20	0.28	0.15		0.15	0.36	0.40	0.09
MODENA	0.13	0.83	0.04		0.83	0.07	0.05	0.06		0.37	0.28	0.28	0.07
NUPACK	0.28	0.72	0.01		**0.39**	0.22	0.14	**0.24**		0.24	0.31	**0.27**	0.18
RNAifold	0.07	0.92	0.02		0.90	0.03	0.04	0.03		0.39	0.27	0.29	0.04

The distribution of nucleotides in paired and unpaired regions are calculated for all existing approaches. Also, the total distribution of nucleotides is presented. The closest values to the natural ones are indicated in bold face.

### Web server

The ERD web server allows biologists to design RNA sequences that fold into a given structure, in an automatic manner. The procedure is fast as most of the requests are completed within seconds. The ERD web server is intuitively arranged with very clear user interface. All the required inputs to run the ERD algorithm should be given in the input form. The target structure must be entered in the dot-parenthesis notation. In this notation, an unpaired base is represented by a dot and a base pair between bases *i* and *j* is represented by a pair of ‘(’ and ‘)’ in position *i* and *j*, respectively.

The constraints over the generated sequences could be also specified as *IUPAC* symbols, where some positions of the generated RNA sequences can be fixed to a specific nucleotides or to a subset of nucleotides. In addition to the sequence constraints, the energy ranges of the generated sequences over the target structure can be determined in ERD web server. This energy range reflects the level of stability for the generated sequences from the thermodynamic point of view. Only some structures with a specific level of energy can take part in certain biological reactions. Therefore, this capability of ERD web server allows researchers to generate sequences with similar structure and a specific level of energy values. This helps us to mimic the biological features of RNA structures that are dependent on the level of their free energies.

**Figure 2:**
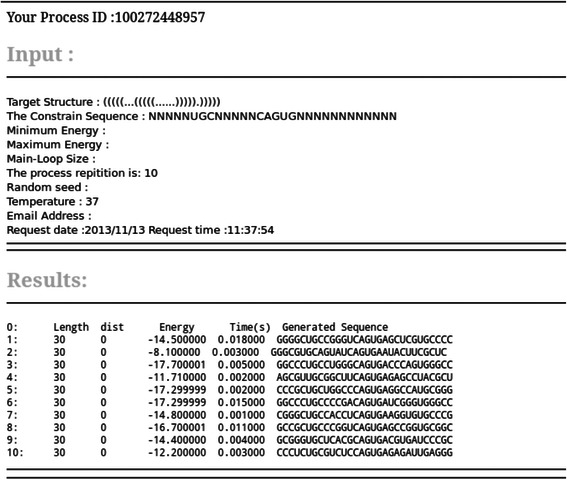
**A typical output of ERD web server.** Here, the target structure and its sequence constraints (fixed nucleotides in internal and hairpin loops) are given as input and 10 RNA sequences are designed with respect to the given constraints.

Finally, the user can choose whether the generated sequences, as well as some additional information, are shown on a web page or send by email. Each job has its own process ID which can be used in the future to retrieve the corresponding results from the server. For all available options, a comprehensive description and detailed examples are provided. The results of a typical computation are presented in Figure 2. On the top of this figure, the input data are summarized. Below that, the length, distances, energy values, execution times, and designed sequences are shown. Additionally, the user can download the results in the form of *FASTA* or *CT* formats.

## Conclusion

We have shown that the ERD tool is a very fast and successful approach to design RNA sequences which fold into a given structure and fulfill some sequence and energy constraints. The core of the algorithm was previously introduced in [5], where we showed that it proceeds better and faster than the other existing approaches. Here, we have demonstrated that the ERD tool, with additional constraints on the sequence and energy level, also performs better and faster than the other competitors.

In addition to the sequence constraints, the energy ranges of the generated sequences over the target structure can be specified in ERD tool. This capability of ERD allows researchers to generate sequences with similar structure and a specific level of energy value.
